# Supplementary material for: Resolving the full spectrum of human genome variation using Linked-Reads
Source: Genome Res. 2019 Apr;29(4):635–45. doi: 10.1101/gr.234443.118 (PMC6442396; doi:10.1101/gr.234443.118)
Supplement: Supplemental Material [file supp_gr.234443.118_Supplemental_longranger-2.2.zip › longranger-2.2.tar.bz2!/lr2.2-public/lib/stan/cmdstan/stan/doc/api/html/term__grammar_8hpp_source.html]

Stan: src/stan/gm/grammars/term\_grammar.hpp Source File


|  |  |
| --- | --- |
|  | Stan  2.5.0  probability, sampling & optimization |


- Main Page
- Related Pages
- Namespaces
- Classes
- Files

- File List
- File Members

All Classes Namespaces Files Functions Variables Typedefs Enumerations Enumerator Friends Macros Pages

- src
- stan
- gm
- grammars

term\_grammar.hpp

Go to the documentation of this file.

1 #ifndef STAN\_\_GM\_\_PARSER\_\_TERM\_GRAMMAR\_\_HPP

2 #define STAN\_\_GM\_\_PARSER\_\_TERM\_GRAMMAR\_\_HPP

3

4 #include <string>

5 #include <sstream>

6 #include <vector>

7

8 #include <boost/spirit/include/qi.hpp>

9

10 #include <stan/gm/ast.hpp>

11 #include <stan/gm/grammars/whitespace\_grammar.hpp>

12 #include <stan/gm/grammars/expression\_grammar.hpp>

13

14 namespace stan {

15

16  namespace gm {

17

18  template <typename Iterator>

19  struct term\_grammar;

20

21  template <typename Iterator>

22  struct expression\_grammar;

23

24  template <typename Iterator>

25  struct term\_grammar

26  : public boost::spirit::qi::grammar<Iterator,

27  expression(var\_origin),

28  whitespace\_grammar<Iterator> > {

29

30  term\_grammar(variable\_map& var\_map,

31  std::stringstream& error\_msgs,

32  expression\_grammar<Iterator>& eg);

33

34  variable\_map& var\_map\_;

35

36  std::stringstream& error\_msgs\_;

37

38  stan::gm::expression\_grammar<Iterator>& expression\_g;

39

40

41  boost::spirit::qi::rule<Iterator,

42  std::vector<expression>(var\_origin),

43  whitespace\_grammar<Iterator> >

44  args\_r;

45

46

47  boost::spirit::qi::rule<Iterator,

48  std::vector<expression>(var\_origin),

49  whitespace\_grammar<Iterator> >

50  dims\_r;

51

52

53  boost::spirit::qi::rule<Iterator,

54  double\_literal(),

55  whitespace\_grammar<Iterator> >

56  double\_literal\_r;

57

58

59  boost::spirit::qi::rule<Iterator,

60  boost::spirit::qi::locals<bool>,

61  expression(var\_origin),

62  whitespace\_grammar<Iterator> >

63  factor\_r;

64

65

66  boost::spirit::qi::rule<Iterator,

67  fun(var\_origin),

68  whitespace\_grammar<Iterator> >

69  fun\_r;

70

71  boost::spirit::qi::rule<Iterator,

72  integrate\_ode(var\_origin),

73  whitespace\_grammar<Iterator> >

74  integrate\_ode\_r;

75

76

77  boost::spirit::qi::rule<Iterator,

78  std::string(),

79  whitespace\_grammar<Iterator> >

80  identifier\_r;

81

82

83  boost::spirit::qi::rule<Iterator,

84  expression(var\_origin),

85  whitespace\_grammar<Iterator> >

86  indexed\_factor\_r;

87

88

89  boost::spirit::qi::rule<Iterator,

90  int\_literal(),

91  whitespace\_grammar<Iterator> >

92  int\_literal\_r;

93

94

95  boost::spirit::qi::rule<Iterator,

96  expression(var\_origin),

97  whitespace\_grammar<Iterator> >

98  negated\_factor\_r;

99

100

101  boost::spirit::qi::rule<Iterator,

102  expression(var\_origin),

103  whitespace\_grammar<Iterator> >

104  exponentiated\_factor\_r;

105

106

107  boost::spirit::qi::rule<Iterator,

108  expression(var\_origin),

109  whitespace\_grammar<Iterator> >

110  term\_r;

111

112

113  boost::spirit::qi::rule<Iterator,

114  variable(),

115  whitespace\_grammar<Iterator> >

116  variable\_r;

117

118  };

119

120  }

121 }

122

123 #endif

stan::gm::term\_grammar::var\_map\_

variable\_map & var\_map\_

**Definition:** term\_grammar.hpp:34

stan::gm::term\_grammar::variable\_r

boost::spirit::qi::rule< Iterator, variable(), whitespace\_grammar< Iterator > > variable\_r

**Definition:** term\_grammar.hpp:116

stan::gm::term\_grammar::term\_r

boost::spirit::qi::rule< Iterator, expression(var\_origin), whitespace\_grammar< Iterator > > term\_r

**Definition:** term\_grammar.hpp:110

expression\_grammar.hpp

stan::gm::int\_literal

**Definition:** ast.hpp:297

stan::gm::expression\_grammar

**Definition:** expression07\_grammar.hpp:23

stan::gm::term\_grammar::int\_literal\_r

boost::spirit::qi::rule< Iterator, int\_literal(), whitespace\_grammar< Iterator > > int\_literal\_r

**Definition:** term\_grammar.hpp:92

stan::gm::term\_grammar::expression\_g

stan::gm::expression\_grammar< Iterator > & expression\_g

**Definition:** term\_grammar.hpp:38

stan::gm::term\_grammar::exponentiated\_factor\_r

boost::spirit::qi::rule< Iterator, expression(var\_origin), whitespace\_grammar< Iterator > > exponentiated\_factor\_r

**Definition:** term\_grammar.hpp:104

stan::gm::term\_grammar::double\_literal\_r

boost::spirit::qi::rule< Iterator, double\_literal(), whitespace\_grammar< Iterator > > double\_literal\_r

**Definition:** term\_grammar.hpp:56

stan::gm::term\_grammar::integrate\_ode\_r

boost::spirit::qi::rule< Iterator, integrate\_ode(var\_origin), whitespace\_grammar< Iterator > > integrate\_ode\_r

**Definition:** term\_grammar.hpp:74

stan::math::integrate\_ode

std::vector< std::vector< typename stan::return\_type< T1, T2 >::type > > integrate\_ode(const F &f, const std::vector< T1 > y0, const double t0, const std::vector< double > &ts, const std::vector< T2 > &theta, const std::vector< double > &x, const std::vector< int > &x\_int, std::ostream \*msgs)

Return the solutions for the specified system of ordinary differential equations given the specified ...

**Definition:** integrate\_ode.hpp:61

stan::gm::double\_literal

**Definition:** ast.hpp:307

stan::gm::term\_grammar::term\_grammar

term\_grammar(variable\_map &var\_map, std::stringstream &error\_msgs, expression\_grammar< Iterator > &eg)

**Definition:** term\_grammar\_def.hpp:625

stan::gm::term\_grammar::fun\_r

boost::spirit::qi::rule< Iterator, fun(var\_origin), whitespace\_grammar< Iterator > > fun\_r

**Definition:** term\_grammar.hpp:69

stan::gm::term\_grammar::args\_r

boost::spirit::qi::rule< Iterator, std::vector< expression >var\_origin), whitespace\_grammar< Iterator > > args\_r

**Definition:** term\_grammar.hpp:44

stan::gm::variable\_map

**Definition:** ast.hpp:439

stan::gm::term\_grammar::error\_msgs\_

std::stringstream & error\_msgs\_

**Definition:** term\_grammar.hpp:36

stan::gm::expression

**Definition:** ast.hpp:216

stan::gm::term\_grammar::identifier\_r

boost::spirit::qi::rule< Iterator, std::string(), whitespace\_grammar< Iterator > > identifier\_r

**Definition:** term\_grammar.hpp:80

ast.hpp

stan::gm::term\_grammar::factor\_r

boost::spirit::qi::rule< Iterator, boost::spirit::qi::locals< bool >, expression(var\_origin), whitespace\_grammar< Iterator > > factor\_r

**Definition:** term\_grammar.hpp:63

stan::gm::fun

**Definition:** ast.hpp:350

stan::gm::whitespace\_grammar

**Definition:** whitespace\_grammar.hpp:11

whitespace\_grammar.hpp

stan::gm::variable

**Definition:** ast.hpp:323

stan::gm::term\_grammar::negated\_factor\_r

boost::spirit::qi::rule< Iterator, expression(var\_origin), whitespace\_grammar< Iterator > > negated\_factor\_r

**Definition:** term\_grammar.hpp:98

stan::gm::term\_grammar::indexed\_factor\_r

boost::spirit::qi::rule< Iterator, expression(var\_origin), whitespace\_grammar< Iterator > > indexed\_factor\_r

**Definition:** term\_grammar.hpp:86

stan::gm::var\_origin

int var\_origin

**Definition:** ast.hpp:411

stan::gm::term\_grammar::dims\_r

boost::spirit::qi::rule< Iterator, std::vector< expression >var\_origin), whitespace\_grammar< Iterator > > dims\_r

**Definition:** term\_grammar.hpp:50


---

[ Stan Home Page ]

*© 2011–2014,
Stan Development Team.*
